# Decreasing incidence of hospital diagnosed CKD/CKDu in North Central Province of Sri Lanka: is it related to provision of drinking water reverse osmosis plants?

**DOI:** 10.1186/s12882-024-03534-w

**Published:** 2024-03-11

**Authors:** Asanga Venura Ranasinghe, Lakshmi C. Somatunga, Gardiye Weligamage Gamini Priyantha Kumara, Ranamuka Henayage Karunarathna, Ambepitiyawaduge Pubudu De Silva, Jayaprakara Mudiyanselage Chathurika Nayani Gunawardena, Sembu Kuttige Champika Ruwan Kumari, Mohamed Shali Fathima Sarjana, Mannikawadumesthri Vipula Chandu De Silva

**Affiliations:** 1 National Renal Disease Prevention and Research Unit, Ministry of Health, Colombo 10, Sri Lanka; 2 Office of Additional Secretary, Ministry of Health, Colombo 10, Sri Lanka; 3 Department of Pathology, Faculty of Medicine, University of Colombo, Colombo 08, Sri Lanka

**Keywords:** (CKDu) Chronic Kidney Disease of uncertain origin, Decreasing incidence of CKD/CKDu, Provision of safe drinking water

## Abstract

**Background:**

We assessed the possible impact of provision of reverse osmosis (RO) water on the incidence of hospital diagnosed CKD/CKDu in North Central Province (NCP) of Sri Lanka.

**Methods:**

An ecological study was conducted on data from 2010–2020 on the incidence of hospital diagnosed CKD/CKDu, CKD/CKDu screening and provision of drinking water RO plants in NCP. Analysis was conducted using descriptive statistics, ANOVA and chi-square test.

**Results:**

The annual incidence of hospital diagnosed CKD/CKDu (per 100 000 population) in 2010–2013, 2014–2016 and 2017–2020 periods in Anuradhapura district were 129.07, 331.06 and 185.57 (*p* = 0.002) while in Polonnaruwa district these were 149.29, 326.12 and 296.73 (*p* = 0.04) respectively. In NCP provision of RO plants commenced after 2011 and the decline in the incidence of hospital diagnosed CKD/CKDu was seen in 25 of the 29 Divisional Secretary Divisions when more than 20% of the families received access to drinking RO water projects.

**Conclusions:**

The annual incidence of hospital diagnosed CKD/CKDu increased in NCP from 2010 to 2016 and continuously decreased thereafter. Continuous declining of CKD/CKDu incidence was seen after more than 20% of the families received access to drinking water RO plants.

## Key message

The incidence of hospital diagnosed CKD/CKDu in CKDu affected North Central Province of Sri Lanka is decreasing possibly due to provision of safe drinking water.

## Introduction

Chronic kidney disease of uncertain origin (CKDu) was first identified in Sri Lanka in early 1990s from Madawachchiya Divisional Secretariat (DS) Division in the Anuradhapura district [1]. Two decades later it was endemic in the North Central Province (NCP) and had spread to bordering districts as well [2]. Despite many studies the cause of CKDu still remains elusive [3–10]. Nevertheless, most of these CKDu causation hypotheses linked drinking water as the source of entry of the injurious agent to the human body [11]. The water in NCP is hard and unpalatable [12, 13] resulting in residents drinking inadequately to quench their thirst [14]. Based on this hypothesis the government commenced provision of reverse osmosis (RO) water [15, 16]. This was supplemented by health education programmes to promote adequate drinking of safe water [17]. A recent publication described a decrease in the incidence of CKDu in NCP possibly due to provision of safe drinking water [2]. In this study we analyzed the possible impact of provision of RO water on the incidence of hospital diagnosed CKD/CKDu in North Central Province of Sri Lanka.

## Methods

This was an ecological study looking at population level data on the incidence of hospital diagnosed chronic kidney disease (CKD)/CKDu, CKD/CKDu screening and provision of RO water in North Central Province. The methodology for assessing the incidence of hospital diagnosed CKD/CKDu in NCP was previously described in detail [2]. To calculate the incidence of hospital diagnosed CKD/CKDu, all newly diagnosed patients were prospectively collected from 11 hospitals on a monthly basis. The hospitals included Teaching Hospitals, District General Hospitals, Base Hospitals and Divisional Hospitals of the Anuradhapura and Polonnaruwa districts. The diagnosis of CKD (which includes CKD and CKDu) and staging of the disease had been made by a Nephrologist or Consultant Physician according to the Kidney Disease: Improving Global Outcomes paper [18]. The GFR estimations were derived using the CKD-EPI creatinine Eq. [19]. As the definitive guidelines for distinguishing CKD from CKDu (in Sri Lanka) according to World Health Organization criteria became available only after 2016 [20], attempts were not made to distinguish between CKD and CKDu. Although an early guideline for case definition of CKDu was available [3] we observed during GIS mapping of CKD/CKDu patients [2] that the Nephrologists/Consultant Physicians in Anuradhapura and Polonnaruwa did not use this guideline and documented most CKDu cases as CKD.

The CKD/CKDu screening information from 2010–2020 was obtained from the Regional Director of Health Services of Anuradhapura and Polonnaruwa districts. The formal organized screening programmes in NCP were initiated in 2014. Screening was conducted using urine albumin to creatinine ratio and serum creatinine. Estimation of urine albumin to creatinine ratio and serum creatinine was done at the nearest hospital laboratory. Urine albumin to creatinine ratio of greater than 30 mg/g or eGFR less than 60 ml/1.73m^2^/min were considered as screening positive and referred to nearest government hospital [21]. CKD/CKDu high incidence DS divisions were given priority, thus high proportions were screened in these areas. In the North Central Province concerning CKD/CKDu, there exist villages with both high and low incidence rates. However, irrespective of the hospital-diagnosed incidence rates at the village level, all individuals were given an equal opportunity to undergo CKD/CKDu screening when it was conducted. There was no prioritization based on age, sex, or any other factor during the screening process.

The information with regard to RO plants were gathered routinely by the National Renal Disease Prevention and Research Unit (NRDPRU) of the Ministry of Health, Sri Lanka. NRDPRU conducts surveys in DS Divisions of NCP to map the drinking water RO plants as provision and management of drinking water RO plants is part of their responsibility. To date more than 1,000 drinking water RO plants have been provided to NCP. Before visiting the DS Division for the drinking water RO plant survey, a map of the CKD/CDKu patients in the area along with main roads was created using ArcGIS. ArcGIS is a Geographic Information System (GIS), which is used to display geographic data and create maps. Information on drinking water RO plant locations were gathered on each Gramaniladhari (GN) division of DS Divisions separately from the existing database of NRDPRU. A GN Division is the smallest administrative division in the country and each DS Division in NCP is made up of more than 25 GN Divisions. During the survey, drinking water RO plants provided and managed through other organizations were searched by interviewing key informant field officers (eg. Gramasewaka, Samurdhi Officer, and Agriculture Officers) and private drinking water suppliers of the GN Division. The operator of the RO plant was also interviewed separately to obtain the required information.

The data gathered was statistically analyzed using STATA 13 student version. Counts and percentages were used to express discrete variables while mean and standard deviation were used to express continuous variables. For analysis purposes we divided the study population into two time segments as follows: the period of increasing incidence of hospital diagnosed CKD/CKDu from 2010 to 2016 and the period of decreasing incidence from 2017 to 2020. The first segment was further divided into two time segments: where CKD/CKDu formal organized screening was not conducted (from 2010 to 2013) and CKD/CKDu formal organized screening had commenced (from 2014 to 2016). To calculate the incidence, population data was obtained from the 2011 Census [22] and expressed as per 100,000 population. All incidence rates calculated were annual rates. The annual hospital diagnosed CKD/CKDu incidence increase rate was calculated by subtracting the incidence of the current year from the previous year.

## Results

During 2010 to 2020 period 362,293 and 205,767 were screened for CKD/CKDu, in Anuradhapura and Polonnaruwa districts respectively. In Anuradhapura district the highest number of population was screened in 2016 (89, 184) and in Polonnaruwa in 2017 (44, 681). Average proportion of population annually screened for CKD/CKDu in both districts was 6.7% (3.1 SD) and 8.5% (2.2 SD) respectively. From 2015 to 2020, In Anuradhapura district, among DS divisions, those with the six highest incidence of hospital diagnosed CKD/CKDu (annual hospital diagnosed CKD/CKDu incidence of > 500 per 100,000 population from 2014 to 2016) three had screened more than 50% of the population while among the six lowest DS divisions (annual incidence of < 185 per 100,000 population from 2014 to 2016) 4 had screened less than 23% of the population. In both districts a higher proportion was screened from 2014 to 2016 period.

There were a total of 30,596 new hospital diagnosed CKD/CKDu patients reported from NCP during the period of 2010 to 2020 (19,378 and 11,218 from Anuradhapura and Polonnaruwa districts respectively). The incidence of hospital diagnosed CKD/CKDu increased from 2013 to 2016 and continuously declined thereafter in both districts. Table 1 compares the selected characteristics of the annual incidence of hospital diagnosed CKD/CKDu, CKD/CKDu screening details and provision of drinking water RO plants in Anuradhapura and Polonnaruwa districts from 2010–2013, 2014–2016 and 2017–2020 period.

**Table 1:** Selected characteristics of the incidence of hospital diagnosed chronic kidney disease / chronic kidney disease of uncertain origin, screening details for chronic kidney disease / chronic kidney disease of uncertain origin and provision of drinking water reverse osmosis plants in Anuradhapura and Polonnaruwa districts from 2010–2016 period and 2017–2020 period

**Characteristics**	**Anuradhapura (total CKD/CKDu patients = 19 378)**				**Polonnaruwa (total CKD/CKDu patients = 11 218)**			
	**2010 to 2013**	**2014 to 2016**	**2017 to 2020**	**Level of Significance**	**2010 to 2013**	**2014 to 2016**	**2017 to 2020**	**Level of Significance**
Total CKD/CKDu cases	4443 (22.9%)	8547 (44.1%)	6388 (33.0%)		2425 (21.6%)	3973 (35.4%)	4820 (43.0%)	
**Mean annual incidence of hospital diagnosed CKD/CKDu (per 100 000 population)**								
District (SD)	129.07 (8.46)	331.06 (50.10)	185.57 (71.09)	*F* = 14.17 *P* = 0.0024	149.29 (34.81)	326.12 (98.80)	296.73 (104.11)	*F* = 4.79 *P* = 0.042
Male population (SD)	175.73 (9.95)	406.73 (60.23)	253.93 (93.29)	*F* = 11.00 *P* = 0.0051	215.41 (44.88)	424.51 (115.41)	374.79 (137.03)	*F* = 3.93 *P* = 0.0649
Female population (SD)	84.57 (9.90)	258.89 (40.55)	120.38 (50.23)	*F* = 20.07 *P* = 0.0008	84.63 (25.30)	229.90 (84.31)	220.40 (72.76)	*F* = 6.24 *P* = 0.0233
< 39 years age group (SD)	14.59 (1.94)	41.05 (6.22)	23.36 (5.24)	*F* = 28.46 *P* = 0.0002	17.31 (11.61)	52.67 (20.88)	40.35 (17.81)	*F* = 4.13 *P* = 0.0585
40–59 years age group (SD)	289.47 (6.71)	708.27 (106.11)	360.11 (156.57)	*F* = 13.76 *P* = 0.0026	281.50 (74.58)	582.92 (178.88)	516.16 (186.96)	*F* = 3.97 *P* = 0.0635
> 60 years age group (SD)	575.68 (67.45)	1559.25 (292.80)	962.95 (350.71)	*F* = 11.99 *P* = 0.0039	695.47 (110.92)	1500.70 (425.02)	1451.66 (489.57)	*F* = 5.52 *P* = 0.0002
Annual average incidence increase rate (SD)	-3.64 (18.38)	86.30 (62.78)	-69.66 (40.56)	*F* = 10.82 *P* = 0.0072	26.10 (39.30)	79.13 (55.81)	-70.67 (44.20)	*F* = 9.38 *P* = 0.0104
No. of DS which showed a declining hospital diagnosed CKD/CKDu incidence (Anuradhapura *n* = 22; Polonnaruwa *n* = 7)	1	6	15		0	0	7	
**CKD/CKDu Screening details**								
Total screened (annual mean) [SD]	-	154 032 (77,016) [21,237.25]	208 261 (52,065.25) [29,998.66]	*F* = 1.05 *P* = 0.3627	-	75 826 (37,913) [1742.31]	129 941 (32,485.25) [10,692.61]	*F* = 0.45 *P* = 0.5373
Screened % from population (annual mean) [SD]	-	14.34% (7.17) [1.09]	22.36% (5.59) [3.60]	*F* = 0.33 *P* = 0.5954	-	16.31% (8.15) [7.41]	29.64% (7.41) [3.59]	*F* = 0.08 *P* = 0.7962
No. of DS divisions with highest annual number screened (Anuradhapura *n* = 22; Polonnaruwa *n* = 7)	0	14	8		0	5	2	
No. DS divisions with highest annual number screened occurring on or after decline of hospital diagnosed CKD/CKDu incidence (Anuradhapura *n* = 22; Polonnaruwa *n* = 7)	0	2	7		0	0	2	
No. of DS divisions with highest annual number screened occurring before decline of hospital diagnosed CKD/CKDu incidence (Anuradhapura *n* = 22; Polonnaruwa *n* = 7)	0	12	1		0	5	0	
**Details of RO plants**								
No. of new RO plants	25	353	378		4	124	111	
No. of new beneficiary families (%)	4743 (2.0%)	74,951 (31.2%)	84,289 (35.1%)	*F* = 2.62 *P* = 0.1329	1060 (1.0%)	25,919 (27.1%)	51,673 (47.6%)	*F* = 4.96 *P* = 0.0397
**Provision of drinking water RO plants to DS Divisions (Anuradhapura *n* = 22; Polonnaruwa *n* = 7)**								
No. of DS which commenced RO projects (%)	9	13	0		2	5	0	
No. of DS with < 20% families getting RO water	22	7	0		7	3	0	
No. of DS with > 20% families getting RO water (%)	0	15	7		0	4	3	
No. of DS with > 20% families getting RO water on or before decline of hospital diagnosed CKD/CKDu incidence	0	6	12		0	0	7	
No. of DS with > 20% families getting RO water after decline of hospital diagnosed CKD/CKDu incidence	1	1	2		0	0	0	

The annual rate of increase of hospital diagnosed CKD/CKDu incidence was highest for Anuradhapura district (158.7) in 2014 while for Polonnaruwa district (143.6) this was observed in 2016. The annual rate of decrease of hospital diagnosed CKD/CKDu incidence was highest for Anuradhapura district in 2017 (105.4) while for Polonnaruwa district this was observed in 2020 (136.4).

**Fig. 1:**
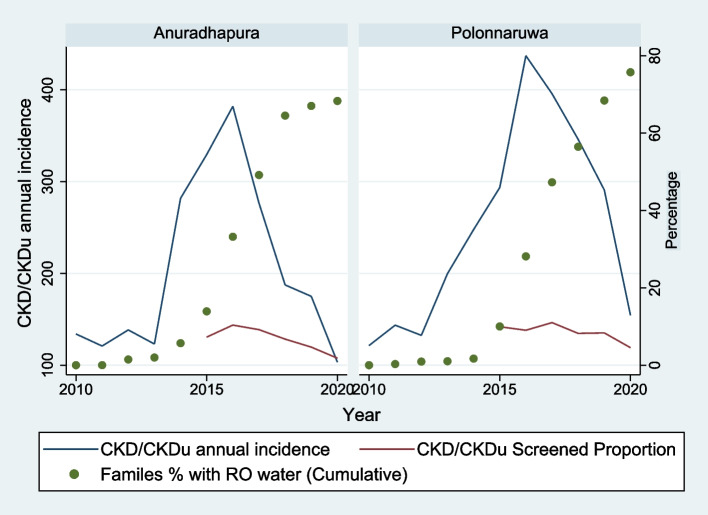
The incidence of hospital diagnosed chronic kidney disease/chronic kidney disease of uncertain origin, screened proportion for chronic kidney disease/chronic kidney disease of uncertain origin and the percentage of families receiving reverse osmosis plant water from 2010 to 2020 in Anuradhapura and Polonnaruwa districts

**Fig. 2:**
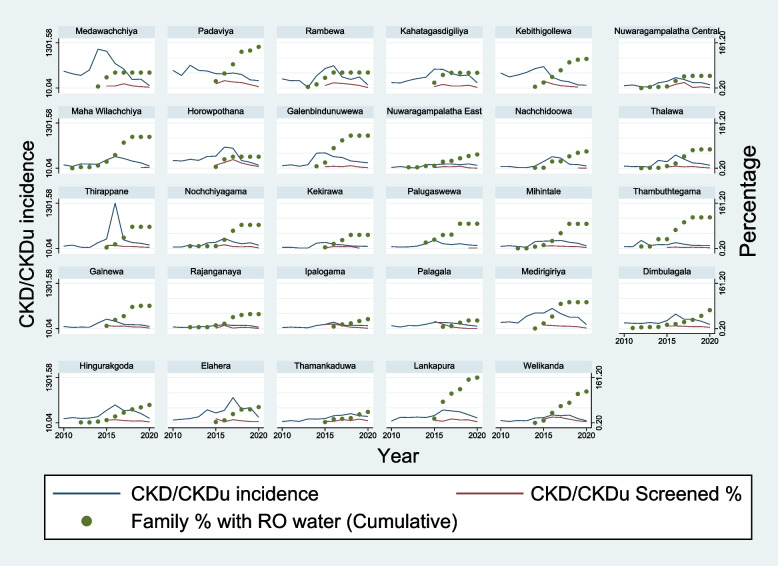
The incidence of hospital diagnosed chronic kidney disease/chronic kidney disease of uncertain origin, screened proportion for chronic kidney disease/chronic kidney disease of uncertain origin and the percentage of families receiving reverse osmosis plant water from 2010 to 2020 in each DS division of North Central Province

Figure 1 shows the incidence of hospital diagnosed CKD/CKDu, CKD/CKDu screening proportion and the percentage of families receiving RO plant water from 2010 to 2020 in Anuradhapura and Polonnaruwa districts and Fig. 2 demonstrates these at each DS divisions of NCP.

**Fig. 3:**
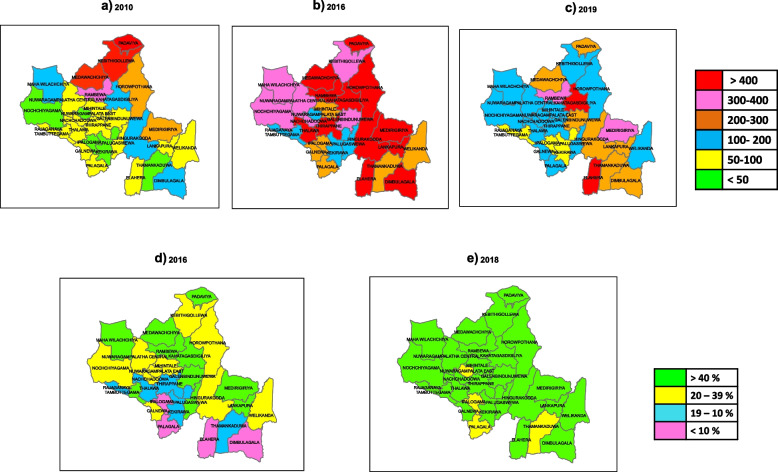
The distribution of incidence of hospital diagnosed chronic kidney disease/chronic kidney disease of uncertain origin in North Central Province for the years (**a**) 2010, (**b**) 2016 & (**c**) 2019 and the availability of RO water in North Central Province for the years (**d**) 2016 and (**e**) 2018

Figure 3 illustrates the distribution of incidence of hospital diagnosed CKD/CKDu in NCP for the years a) 2010, b) 2016 & c) 2019 and the availability of RO water in d) 2016 and e) 2018.

## Discussion

The occurrence of CKD/CKDu has previously shown a clustering pattern (Fig. 3) with highly endemic areas being clustered closely together [2]. Areas with a high incidence of hospital diagnosed CKD/CKDu are located mainly within the NCP together with DS divisions of adjacent districts [2, 23]. The incidence of hospital diagnosed CKD/CKDu decreases gradually from the epicenter of high incidence areas [2]. This may be because environmental factor/factors contributing to CKDu reduce gradually when moving away from the epicenter of high CKD/CKDu incidence areas.

The incidence of hospital diagnosed CKD/CKDu in Anuradhapura and Polonnaruwa districts increases from 2010 to 2016 and thereafter decreases (Fig. 1). The incidence of hospital diagnosed CKD/CKDu in all DS divisions of Anuradhapura and Polonnaruwa districts demonstrate a more or less similar trend (Fig. 2).

During the early period of our study a high proportion of the diagnosed CKD would have been CKDu. With the prevalence of diabetes increased to 16.5% in NCP [24] and the prevalence of hypertension increased to 26% in Anuradhapura district [25] towards the later part of our study, a significant portion of CKD in NCP may be related to hypertension and diabetes, in comparison to 2009 when the first paper on CKDu was published [26]. Hence, the possibility of a significant dilution of the study sample by diabetes/hypertension-associated CKD must be acknowledged. In this view the actual CKD figures towards latter part of the study should increase considering the increased prevalence rates of diabetes and hypertension. Therefore, this may also indicate the declining incidence is actually involves the CKDu proportion. As CKD due to diabetes and hypertension takes a long time it is possible that there may be an increase in CKD rates in another 5 to 10 years time.

Most hypotheses for the causation of CKDu identified water as the medium of toxin entry into the human body [11]. This knowledge and poor accessibility to safe drinking water in the affected areas, led to acceleration of provision of safe drinking water to NCP by the government [15, 16]. The provision of community based drinking water RO plants commenced after 2011 in NCP [15, 16]. From 2010–2013 period 11 DS Divisions has commenced drinking water RO plant projects and in all of these the decline in the incidence of hospital diagnosed CKD/CKDu occurred after more than 20% of the families received access to drinking RO water projects. The rest of 18 DS Divisions has commenced drinking water RO plant projects during 2014–2016 period and in 14 of these the decline in the incidence of hospital diagnosed CKD/CKDu occurred after more than 20% of the families received access to drinking RO water projects. In four DS divisions of Anuradhapura district decline begins well before reaching “20% family target” probably because these communities have started to access safe drinking water in different formats. Further these were CKD/CKDu low incidence areas (all four areas reporting the highest hospital diagnosed CKD/CKDu incidence rate of less than 215 per 100,000 population) and the residents were socio economically higher compared to other DS divisions. They may have started purchasing safe drinking water before commencement of drinking water RO plants. The initiation of community-based drinking water RO plants were late in these areas and also the number of coverage of drinking water RO plants are less in these areas probably indicating less requirement for community based water projects due to their accessibility to safe drinking water.

The coverage of families accessing drinking water RO plants exceeds 100% in some of the DS divisions as some families were accessing more than one drinking water RO plant.

In 2014 formal organized CKD/CKDu screening commenced in NCP. It is important to note that screening areas were priorities on the CKD/CKDu incidence. High proportions were screened in high incidence areas while low proportions were screened in low incidence areas. The increase in the incidence of hospital diagnosed CKD/CKDu may be attributed to initiation of formal organized CKD/CKDu screening programmes, however the increase in the incidence continues even after the annual proportion being screened drops in most of the DS divisions, including the high incidence DS divisions. When comparing the annual screening rates from 2014–2016 period with 2017–2020 period there was no significant difference (*p* = 0.36 in Anuradhapura district and *p* = 0.54 in Polonnaruwa district) further indicating the trend in hospital diagnosed CKD/CKDu incidence not related to the CKD/CKDu annual screening rates. This concludes that there had been a general increase in the incidence of hospital diagnosed CKD/CKDu from 2013–2016 period and thereafter a rapid continuous decline. Thus, the screening rate and incidence of CKD/CKDu does not seems to have a relationship. Nevertheless, ascertainment bias exists since a direct comparison of hospital diagnosed CKD/CKDu incidence with CKD/CKDu screened population is not possible.

There may be several reasons for the declining trend in hospital diagnosed CKD/CKDu incidence in NCP. Water may have been contaminated or there may have been some inherent factor which was causing the CKDu but aggravated by contaminated water. Thus, provision of safe drinking water may remove the risk of CKDu. Another hypothesis would be the provision of safe drinking water is palatable compared to hard water in the area thus an increase consumption of water due to palatability may have reduced the incidence of hospital diagnosed CKD/CKDu. At the same time, we also wish to draw attention to the fact that some putative nephrotoxins (paraquat, alachlor, chlorpyriphos, propanil and glyphosate) were banned in Sri Lanka from 2011 to 2015. When considering the implications of an environmental toxin, it is worth exploring the influence that this may have on the reduction in incidence of hospital diagnosed CKD/CKDu from 2016 onwards.

Presently the natural history of CKDu is poorly understood, although several theories have been proposed. These include heavy metals, agrochemicals, hardness of the water and cyanotoxins [3–10]. Majority of publications have indicated that the medium of transport for the nephrotoxin is water [11]. Our findings support previous reportings [11, 27] indicating that provision of safe drinking water by supply of RO plants was associated with a decrease in the incidence of hospital diagnosed CKD/CKDu. However, if the nephrotoxin was transmitted by water, the relatively short period of 3.2 years taken to reduce the incidence is difficult to explain because the known mechanisms of renal damage due to nephrotoxins is often due to a chronic tubulointerstitial nephritis [28–30], which would have taken a longer period to reduce the incidence. Although not proven our explanation for this is as follows: The duration of the time taken to manifest the disease may depend on the concentration level of the nephrotoxin absorbed into the body. This may explain the geographical distribution of the disease incidence as the high disease incidence areas may have had a higher concentration of the nephrotoxin with the low incidence areas having a lower concentration. The accumulated nephrotoxin may require a threshold level to cause clinically manifest renal damage with different individuals having different threshold levels to cause the disease. Therefore, provision of safe drinking water midway during the pathogenesis of the disease may have prevented the threshold being reached in some susceptible individuals thereby reducing the incidence [11, 27]. It has been reported that most of the drinking water in NCP is high in hardness and is not palatable resulting in poor hydration levels [12–14]. In fact, in Central America some studies have proposed chronic dehydration and heat stress as a possible mechanism which contributes to CKDu [31, 32]. If provision of palatable drinking water with the supply of RO plants resulted in better hydration of susceptible individuals in NCP of Sri Lanka, there is a possibility that provision of adequate hydration would have contributed to the reduction in incidence. It is also important to note that the regional health authorities of NCP publicised awareness programmes for consumption of safe drinking water which may have resulted in some individuals seeking access to safe drinking water even before community based RO plants were introduced [11, 17]. Although we have no data to support this hypothesis it may explain why the incidence decreased in a short period after introduction of RO plants.

The reduction of the hospital diagnosed CKD/CKDu incidence does not necessarily mean that the water is the transporter of the cause. The provision of safe drinking water probably resulted in a behavioral change in residents of CKDu affected areas to consume more water [15–17] which may have also reduced the risk for the disease. CKDu has also been reported in Central America [33], Andhra Pradesh in India [34], Tierra Blanca in Mexico [35], El-Minia Governorate in Egypt [36] and in Tunisia [37]. The arguments for the causation of CKDu in these areas are more or less similar compared to the Sri Lankan situation with high emphasis on contaminated water and occupational driven heat stress [38–41]. It would be noteworthy to assess the impact of provision of safe drinking water on incidence of CKDu in these areas.

## Conclusion

In Anuradhapura and Polonnaruwa districts of NCP an increase in the incidence of hospital diagnosed CKD/CKDu was observed from 2010 to 2016 followed by a decreasing trend since 2017. Similar trend in the incidence of CKD/CKDu was seen in all DS divisions of Anuradhapura and Polonnaruwa districts as well. Provision of drinking water RO plants commenced in 2011 in NCP targeting CKD/CKDu high incidence areas. Continuous declining of hospital diagnosed CKD/CKDu incidence was observed after more than 20% of the families received access to drinking water RO plants in Anuradhapura and Polonnaruwa districts. This declining trend may be associated with provision of drinking water RO plants.

## Data Availability

We have provided the raw aggregate data we used for analysis in Table 2, which is also available at the renal registry https://nicst.com/iframe-renal-dev/. The incidence information is also published in the Sri Lanka Annual Health Bulletin 2021. However, if anyone requires the same raw aggregate data they can be obtained from the National Renal Disease Prevention and Research Unit, Ministry of Health, Sri Lanka.

**Table 2:** Incidence of hospital diagnosed chronic kidney disease/chronic kidney disease of uncertain origin, number screened for chronic kidney disease/chronic kidney disease of uncertain origin and the percentage of families provided with reverse osmosis water from 2010 to 2020 in Anuradhapura and Polonnaruwa districts

District/Division	Indicator	2010	2011	2012	2013	2014	2015	2016	2017	2018	2019	2020
**Anuradhapura district**	CKD/CKDu incidence	134.0	120.8	138.4	123.1	281.8	329.4	381.9	276.6	187.4	175.0	103.3
	% Families receiving RO water			1.5	2.0	5.7	13.9	33.2	49.2	64.5	67.0	68.3
	No. Screened						62,427	89,184	79,051	57,957	40,172	15,586
Medawachchiya	CKD/CKDu incidence	492.5	420.0	373.1	528.7	1119.3	1063.8	709.9	556.4	251.6	266.5	78.9
	% Families receiving RO water					5.9	38.3	55.2	55.2	55.2	55.2	55.2
	No. Screened						3513	3420	7221	3753	2484	1479
Padaviya	CKD/CKDu incidence	587.0	417.4	756.6	587.0	565.3	478.3	469.6	500.0	452.2	273.9	247.8
	% Families receiving RO water						24.7	52.0	84.7	129.5	133.8	146.9
	No. Screened						3525	6052	4998	4562	2854	1224
Rambewa	CKD/CKDu incidence	304.5	244.7	250.1	24.5	405.1	647.1	742.2	367.0	288.2	367.0	103.3
	% Families receiving RO water				4.2	13.3	36.6	55.5	55.5	55.5	55.5	55.5
	No. Screened						3457	6946	6056	4992	3414	737
Kahatagasdigiliya	CKD/CKDu incidence	181.0	171.1	252.9	317.3	357.0	629.7	622.2	488.4	409.0	433.8	178.5
	% Families receiving RO water						19.1	46.8	54.4	54.4	54.4	54.4
	No. Screened						2155	5240	3031	2976	4050	1090
Kebithigollewa	CKD/CKDu incidence	497.2	371.8	434.5	519.6	645.0	712.2	362.8	250.8	206.0	107.5	94.1
	% Families receiving RO water					4.8	19.9	39.7	63.4	91.7	101.2	104.1
	No. Screened						5645	3395	1914	1351	806	
NPC (Nuwaragampalatha Central)	CKD/CKDu incidence	78.4	102.9	44.1	37.6	176.4	223.8	347.9	284.2	184.6	179.7	114.3
	% Families receiving RO water			0.4	3.8	4.2	6.2	25.6	41.7	43.6	43.6	43.6
	No. Screened						1975	8118	12,434	1444	2984	847
Maha wilachchiya	CKD/CKDu incidence	106.8	62.3	133.5	129.8	124.6	249.3	342.7	289.3	213.6	164.7	75.7
	% Families receiving RO water		0.2	4.1	4.1	9.7	23.5	46.9	91.3	111.8	111.8	111.8
	No. Screened										518	665
Horowpothana	CKD/CKDu incidence	256.8	240.6	294.7	262.2	392	400.1	700.2	665.0	256.8	197.4	102.7
	% Families receiving RO water						5.1	31.9	41.1	41.1	41.1	41.1
	No. Screened						4186	7540	11,480	6860	4201	2234
Galenbindunuwewa	CKD/CKDu incidence	80.9	114.9	66.0	117.0	500.1	508.6	385.2	355.4	242.6	210.7	180.9
	% Families receiving RO water					7.1	20.5	71.7	99.3	116.3	116.3	116.3
	No. Screened						2716	3303	2712	4275	2016	887
NPE (Nuwaragampalatha East)	CKD/CKDu incidence	25.8	57.4	21.5	10.0	114.7	121.9	137.7	142.0	120.4	143.4	97.5
	% Families receiving RO water			3.5	3.5	8.0	8.6	23.3	26.4	35.5	43.5	49.1
	No. Screened						1724	3858	3617	5019	992	278
Nachchidoowa	CKD/CKDu incidence	63.0	59.1	23.6	19.7	74.9	224.6	382.2	338.9	134.0	122.2	74.9
	% Families receiving RO water					1.7	1.7	24.1	24.9	42.4	55.0	59.9
	No. Screened										168	152
Thalawa	CKD/CKDu incidence	72.7	55.4	62.3	36.3	261.3	216.3	437.8	299.3	178.2	154.0	98.6
	% Families receiving RO water			0.6	1.7	1.7	8.0	17.5	42.4	64.5	67.3	67.3
	No. Screened						2812	4523	2923	3706	1957	662
Thirappane	CKD/CKDu incidence	74.0	103.5	44.4	33.3	173.8	277.3	1301.6	251.4	177.5	155.3	107.2
	% Families receiving RO water						3.3	14.7	38.2	76.9	76.9	76.9
	No. Screened						2771	2835	1899	1899	1307	980
Nochchiyagama	CKD/CKDu incidence	46.1	48.1	110.2	52.1	182.4	202.5	332.8	218.5	152.3	186.4	106.2
	% Families receiving RO water			8.0	8.0	8.0	8.4	30.9	62.8	83.7	83.7	83.7
	No. Screened						3775	6385	3358	2761	3483	893
Kekirawa	CKD/CKDu incidence	37.1	35.4	11.8	11.8	173.9	200.9	138.4	118.2	82.7	72.6	67.5
	% Families receiving RO water						3.9	16.1	29.6	48.1	48.1	48.1
	No. Screened						1259	3928	3495	3928	558	
Palugaswewa	CKD/CKDu incidence	57.8	44.9	51.3	77.0	154.0	276.0	154.0	128.4	154.0	115.5	96.3
	% Families receiving RO water					20.7	31.2	47.8	49.1	87.9	87.9	87.9
	No. Screened										245	194
Mihintale	CKD/CKDu incidence	68.0	87.8	65.2	36.8	223.8	240.8	246.5	266.3	204.0	170.0	85.0
	% Families receiving RO water			0.4	0.4	6.3	11.6	26.5	54.3	87.5	87.5	87.5
	No. Screened						2962	3175	2774	2433	2321	957
Thambuthtegama	CKD/CKDu incidence	61.3	54.2	263.9	99.0	124.9	120.2	190.9	139.03	101.3	94.3	91.9
	% Families receiving RO water			7.2	7.2	33.0	33.0	65.8	93.1	110.7	110.7	110.7
	No. Screened						1544	2122	1365	1903	1480	1100
Galnewa	CKD/CKDu incidence	69.1	43.2	51.8	48.9	169.8	279.1	218.7	132.4	120.8	115.1	71.9
	% Families receiving RO water						9.7	30.7	44.4	77.0	81.1	81.1
	No. Screened						3573	2904	3099	2088	1868	538
Rajanganaya	CKD/CKDu incidence	53.7	35.8	41.7	56.6	71.6	101.4	131.2	101.4	107.3	95.4	44.7
	% Families receiving RO water			5.0	5.0	5.0	11.4	17.4	40.6	48.3	51.4	51.4
	No. Screened						455	3726	575	1363	1103	122
Ipalogama	CKD/CKDu incidence	30.9	48.9	36.0	25.7	100.4	139	213.6	110.6	102.9	102.9	95.2
	% Families receiving RO water							7.3	14.7	18.7	26.7	33.5
	No. Screened						5614	6366	4129	1875	1280	463
Palagala	CKD/CKDu incidence	94.1	52.9	100.0	91.2	141.2	202.9	205.9	185.3	155.9	108.8	79.4
	% Families receiving RO water							6.4	9.0	20.8	28.9	28.9
	No. Screened						8766	5348	1971	769	83	84
**Polonnaruwa district**	CKD/CKDu incidence	121.4	143.6	132.5	199.7	247.7	293.5	437.1	395.7	346.0	290.8	154.4
	% Families receiving RO water		0.3	0.9	1.0	1.7	10.0	28.1	47.3	56.5	64.0	75.7
	No. Screened						40,345	36,681	44,681	33,362	33,893	18,356
Medirigiriya	CKD/CKDu incidence	204.4	221.1	184.5	408.7	517.0	513.9	666.4	494.1	378.2	376.7	132.7
	% Families receiving RO water					0.4	18.5	42.8	88.0	94.0	94.0	94.0
	No. Screened						8332	6927	5891	5526	4169	1162
Dimbulagala	CKD/CKDu incidence	189.6	175.7	167.0	200.8	169.5	263.6	479.5	308.8	311.3	242.3	136.8
	% Families receiving RO water		2.2	4.8	5.5	6.2	13.6	16.3	23.3	31.2	45.3	65.8
	No. Screened						7306	7364	6878	5355	5573	3782
Hingurakgoda	CKD/CKDu incidence	129.1	160.2	138.4	146.2	188.2	368.6	521.1	364.0	371.8	278.4	140.0
	% Families receiving RO water			1.7	1.7	4.6	10.0	22.9	36.3	48.1	55.4	63.7
	No. Screened						6245	7135	5534	4220	4645	2192
Elahera	CKD/CKDu incidence	91.1	118.4	143.5	202.7	432.6	330.2	414.4	838.0	453.1	505.5	184.4
	% Families receiving RO water						3.0	9.1	31.5	47.4	48.0	56.3
	No. Screened						6599	1779	4912	3276	2095	2098
Thamankaduwa	CKD/CKDu incidence	43.7	74.0	49.7	129.8	125.0	142.0	235.4	254.8	304.5	236.6	213.5
	% Families receiving RO water						2.9	13.1	14.2	16.9	30.2	39.2
	No. Screened						3969	4349	9894	6772	10,976	6160
Lankapura	CKD/CKDu incidence	63.1	181.1	175.6	192.0	175.6	241.4	428.0	397.8	364.9	266.1	164.6
	% Families receiving RO water						14.8	75.1	104.3	120.0	154.2	161.2
	No. Screened						3258	2140	5083	3780	3837	1473
Welikanda	CKD/CKDu incidence	77.0	47.4	80.0	68.1	159.9	171.8	263.5	236.9	257.6	142.1	71.1
	% Families receiving RO water					0.2	8.7	35.8	59.8	71.6	103.1	111.7
	No. Screened						4636	6987	6489	4433	2598	1489
CKD/CKDu incidence		highest proportion										
		Lowest proportion										
		Significant decreasing point										
% Families receiving RO water reaching 20%—40%												
Highest number screened												
Overall CKD/CKDu decreasing year taken as 2017					**2017** Incidence of hospital diagnosed chronic kidney disease/chronic kidney disease of uncertain origin, number screened for chronic kidney disease/chronic kidney disease of uncertain origin and the percentage of families provided with reverse osmosis water from 2010 to 2020 in Anuradhapura and Polonnaruwa districts
